# Porcine proliferative enteropathy: overview of disease dynamics and non-antibiotic alternatives for prevention and control strategies

**DOI:** 10.3389/fvets.2025.1596316

**Published:** 2025-11-07

**Authors:** Luis-Miguel Gómez-Osorio, Felipe Penagos-Tabares, Jasna Bosnjak-Neumuller, Roberto Mauricio Carvalho Guedes, Marko Vasiljevic, Tobias Steiner, Steven McOrist

**Affiliations:** 1 CIBAV Research Group, Faculty of Agrarian Sciences, School of Veterinary Medicine, University of Antioquia, Medellín, Colombia; 2 Patent Co. DOO, Mišićevo, Serbia; 3 Agromed Austria GmbH, Kremsmünster, Austria; 4 Laboratory of Molecular Pathology, Department of Veterinary Clinic and Surgery, Veterinary School, Federal University of Minas Gerais, Belo Horizonte, Brazil; 5 Scolexia Pty Ltd, Moonee Ponds, VIC, Australia

**Keywords:** ileitis, gut health, essential oils, *Lawsonia intracellularis*, lysozyme, niacinamide, phytogenics

## Abstract

Porcine proliferative enteropathy caused by the intracellular bacterium *Lawsonia intracellularis* remains an economically significant health concern in global pig farming. Clinical and subclinical forms of the disease commonly occur, resulting in substantial productivity losses due to effects on pig growth rate, feed efficiency, and mortality. Current management and control strategies rely primarily on effective vaccines and antibiotics. However, due to antimicrobial resistance being a global public health issue, there is a growing interest in and the need for research, development and large-scale implementation of novel and promising alternatives to antibiotics in animal production. This review integrates current research on novel prevention and management strategies, including current trends in phytotherapy (e.g., phytogenic feed additives), probiotics, prebiotics, immunomodulators, advanced vaccination protocols, and genetic resistance trends in swine. This review also discusses the implementation of biosecurity measures, cost-effectiveness, economic implications, and future perspectives of these strategies.

## 1 Introduction

Proliferative enteropathy (PE), also known as ileitis, is an infectious disease affecting pigs and other animals (like horses, rodents, rabbits and deer). It is caused by *Lawsonia intracellularis*, an obligately intracellular Gram-negative, microaerophilic, vibrioid-shaped bacterium. It was first isolated in pure co-culture from infected pig intestines in 1993 using cell culture techniques, with challenge exposure studies then fulfilling Koch’s postulates (1, 2). PE is characterised by a distinctive thickening of the intestinal mucosa due to the proliferation of immature intestinal crypt epithelial cells (3). Lesions often occur in the ileum but may also be seen in the jejunum, cecum and colon. The extent of the lesions determines the clinical status of affected pigs. Mild to severe grey-green sloppy diarrhoea and noticeable weight loss are the main signs in clinically affected animals. Subclinical infection is a common condition in which infected pigs have noticeable retardation in growth rates but lack prominent diarrhoea (4, 5).

PE presents diagnostic challenges in live animals due to its varied clinical manifestations and the nature of the causative pathogen (6). Often, the disease ranges from acute to chronic forms, exhibiting signs such as diarrhoea, reduced weight gain, and in severe cases, mortality (7). The asymptomatic carriers and subclinical infections may further complicate diagnosis. Accurate and timely identification of PE is critical for effective management and control of the disease within swine populations (8). This requires a combination of clinical evaluation, laboratory testing, and an understanding of herd history and management practises. Laboratory diagnostics play a pivotal role in confirming PE (9).

This review aims to provide a comprehensive and up-to-date synthesis of current knowledge on *L. intracellularis* infection in swine, with a particular focus on porcine PE. This article also includes an overview of the global epidemiological relevance of the disease, an exploration of the main risk factors, pathogenesis, clinical manifestations, and diagnostic approaches, as well as host immune responses. Given the increasing global emphasis on reducing antibiotic use in livestock production, the review also critically evaluates non-antibiotic alternatives for preventing and controlling of PE. These include vaccination strategies, nutritional interventions (such as prebiotics, probiotics, phytogenics, feed strategies and nanoparticles), and biosecurity as well as husbandry practises.

## 2 Global relevance

PE is considered endemic in domestic pig herds worldwide, regardless of whether they are raised indoors or outdoors. Surveys have indicated that over 95% of commercial pig herds globally are infected with *L. intracellularis*, as detected by serology and/or faecal PCR assays (10, 11). The prevalence at the herd level, as reported in multiple studies across various countries, ranged between 48 and 100% (see Table 1). Transmission of *L. intracellularis* occurs by the faecal-oral route from infected pigs and the contaminated environment to susceptible pigs. The level of faecal shedding can be high in some infected pigs, and the infective dose is considered relatively low (10^3^ organisms of *L. intracellularis*/g of faeces). Previous studies of infected pigs have shown a faecal burden range of 10^4^ to 10^8^ *L. intracellularis*/g of faeces (via qPCR detection), with a lower limit of detection of 10^3^ *L. intracellularis*/g of faeces (12).

**Table 1:** A summary of prevalence studies conducted worldwide since 2000, as documented in peer-reviewed articles.

Region	Sample	Methodology	Herd prevalence (%)	References
Europe	Faeces/blood (of nursery, growing and finishing pigs)	qPCR/ELISA	90.3	(10)
Germany			91.7/90	
Denmark			95.8/100	
Spain			83.3/90.0	
France			79.2/100	
The Netherlands			91.7/100	
UK			100/70.0	
Denmark	Faeces (of pigs weighing 30-50 kg)	PCR	93.7	(13)
Sweden	Faeces and rectal swabs (of pigs aged 8–12 weeks)	nPCR	48	(181)
Korea	Blood (of weaning, growing and finishing pigs)	IFA	100	(182)
United States	Blood	ELISA	90.9	(183)
France	Blood	ELISA	88	(16)
Spain	Blood	ELISA	89	(16)
Great Britain and Ireland	Blood	ELISA	92.9–93.1	(22)
Brazil	Blood	IPMA	100	(168)
Thailand	Blood	ELISA	83.3	(184)
Australia	Blood	ELISA	100	(185)
China	Faeces (of sows and fattening pigs)	PCR	93.6	(186)
Chile	Blood	ELISA	100	(187)
Vietnam	Faecal swabs	qPCR	100	(26)

PCR, polymerase chain reaction; nPCR, Nested PCR; RS, rectal swab; IPMA, Immunoperoxidase monolayer assay; IFA, indirect immunofluorescence antibody technique; ELISA, enzyme-linked immunosorbent assay.

## 3 Main risk factors

Pigs of all ages are considered susceptible to *L. intracellularis* infection. On single-site farms with a continuous pig flow between different pig ages/farm areas/housing locations, infection usually occurs a few weeks after weaning, presumably when maternal antibodies fade. This dynamic can be delayed by using effective antimicrobial agents in the nursery area, such as quinoxaline, during the first weeks post-weaning (13–15), forestalling early infections. In this case, clinical disease is usually later in the growing and finishing phases (8). In multi-site farm systems with a distinct separation of groups of post-weaning and breeding pigs by age and site, *L. intracellularis* infection is typically delayed in the grower-finishers until they reach 16 to 20 weeks of age (16). The disease occurs rarely in breeding stock due to age-based immunity and prior exposure and/or vaccination (17, 18). The environment of most pig farms likely contains a sustained level of *L. intracellularis* in the residual faecal material and organic materials in buildings, equipment, or other fomites (19–21). Transmission of faecal matter from contaminated areas to different farm areas, such as those containing breeding animals, would be expected to occur more commonly on single-site farms via boots, rodents, or other fomites (21, 22). Additionally, Musca (house flies) and Eristalis (hover flies) are the farm insects with the highest potential to carry and spread Porcine *L. intracellularis* because parts of their life cycle are closely linked to pigs. Since adult *M. domestica* flies can travel between farms located up to 7 km apart, there is a risk of mechanical transmission of *L. intracelularis* from one farm to another through these insects (23).

**Figure 1:**
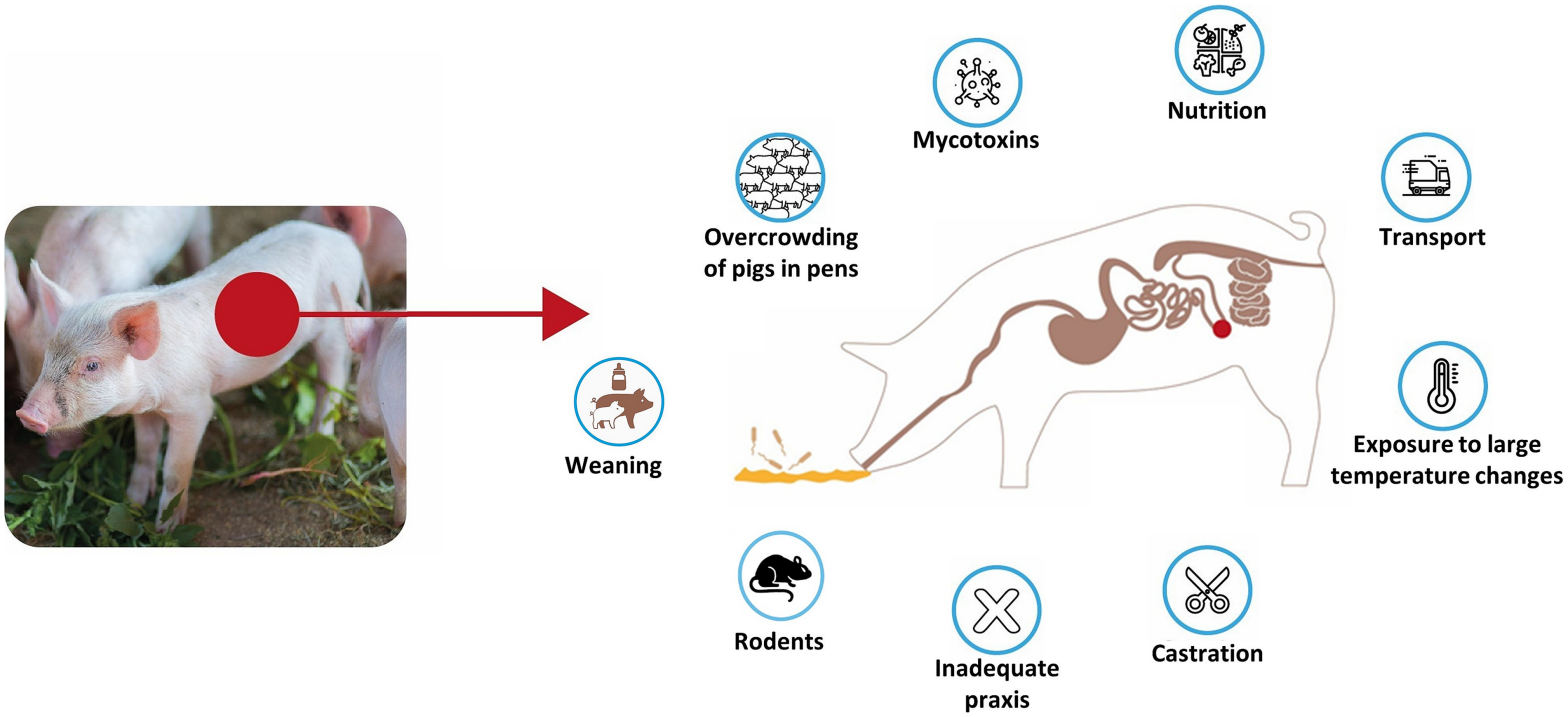
Overview of predisposing stressors and environmental factors contributing to *Lawsonia intracellularis* infection in pigs.

The occurrence of this disease is influenced not only by pathogen exposure but also by a range of environmental and management-related stressors that compromise gut health and immune resilience. Stressful conditions such as weaning, overcrowding, abrupt dietary changes, poor nutritional quality, mycotoxicosis, and inadequate hygiene can induce intestinal barrier damage and increase susceptibility to infection (24, 25). Additionally, procedural stressors such as castration, transport, and sudden temperature fluctuations exacerbate immunosuppression and may increase pathogen shedding and transmission. The presence of rats is also considered a risk factor for PE (26–28). These factors collectively disturb the gut microbiota, weaken mucosal defences, and may facilitate colonisation by *L. intracellularis,* particularly in weaned piglets undergoing rapid physiological and environmental transitions. An overview of these stressors and their possible impact on PE susceptibility is summarised in Figure 1. Recent studies suggest that transmission of *L. intracellularis* may begin before weaning. For example, *L. intracellularis* has been detected in 3-week-old piglets using molecular techniques in ileal digesta and mucosal scrapings, indicating colonisation during lactation or via maternal or environmental sources (19). Whilst direct evidence of vertical (in utero) transmission remains limited, these findings underscore the potential for mother/progeny transmission (either passive immunity or early active exposure), which may be critical in designing preventive and control measures (19).

## 4 Pathogenesis

**Figure 2:**
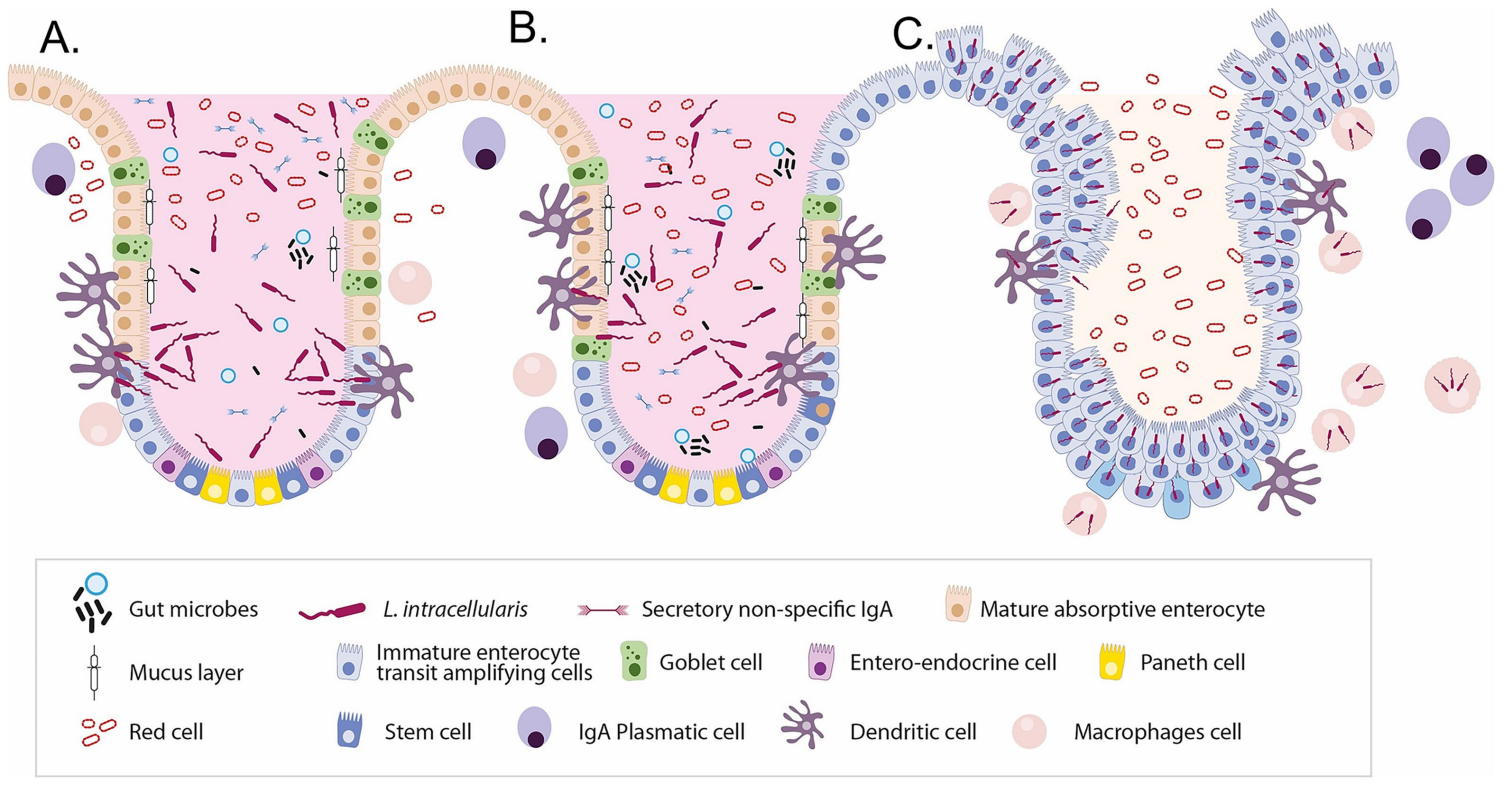
Sequential alterations in intestinal crypt cellular composition and immune responses during *Lawsonia intracellularis* infection. **(A)** Normal porcine intestinal crypt showing a well-organised architecture with balanced epithelial cell types, a protective mucus layer, commensal microbes, and local immune cells. **(B)** Early infection phase: *L. intracellularis* invades immature enterocytes, leading to inflammation, disruption of normal epithelial turnover, and early loss of goblet cells. **(C)** Advanced infection: Extensive crypt hyperplasia and the dominance of proliferating immature enterocytes result in loss of cellular diversity, mucin depletion, immune dysregulation, and compromised barrier function. These changes underlie the characteristic histopathology of proliferative enteropathy.

*L. intracellularis* infects the small and large intestines, leading to PE or proliferative haemorrhagic enteritis. The pathogenesis involves infection of epithelial cells, with the bacteria spreading as these cells divide and migrate (29). Pathogenesis involves the replication of *L. intracellularis* in the cytoplasm of enterocytes, leading to cell maturation failure and ultimately resulting in proliferative enteropathy (Figure 2). Most pigs eventually recover, and cellular immunity is believed to play an essential role in disease resolution (30, 31).

The intestinal epithelium is the second largest epithelium in the pig, after its lungs and is characterised by its rapid self-renewal and dynamic function (32, 33). This tissue undergoes complete regeneration every 2 to 3 days, making it the fastest-renewing tissue in the body (34–36). This rapid turnover is essential for maintaining barrier integrity and ensuring efficient nutrient absorption (37, 38). The renewal process originates in the intestinal crypts, where stem cells, including actively dividing leucine-rich repeat-containing G-protein coupled receptor 5 (LGR5^+^, used as a stem cell marker) crypt base columnar cells and quiescent label-retaining cells (LRCs) at the +4 position, give rise to transit-amplifying progenitor cells (36, 39). These cells migrate upward along the crypt–villus axis, differentiating into absorptive enterocytes and various secretory cell types, including goblet cells, enteroendocrine cells, microfold (M) cells, and Paneth cells. However, the latter are rare in pigs (40, 41).

In newborn piglets, the gastrointestinal tract is relatively immature, making these animals very susceptible to infectious diseases. Additionally, after some weeks, weaning is also a critical period due to the multiple stressors it introduces, such as transient anorexia, intestinal inflammation, and unbalanced gut microbiota (24, 42). An overview of the dynamics of intestinal crypts, emphasising the critical role of crypt-villus architecture in both nutrient uptake and serving as a barrier, is mandatory to understand PE comprehensively (33). Continuous cell turnover is a characteristic of the intestinal epithelium, fuelled by stem cells at the crypt base. These stem cells are supported and safeguarded by a niche comprising specialised cells, which also dictates competition for space and influences cell fate.

There are various signalling pathways, including Wnt (Wingless and Int-1), Neurogenic locus notch homologue (Notch), epidermal growth factor (EGF) and bone morphogenetic protein (BMP), which are integral to maintaining stem cell populations and guiding differentiation. Any imbalances in these pathways can lead to disease, showing the delicate balance required for intestinal homeostasis and the complex interactions within the crypt niche (33, 34).

The intestinal epithelium undergoes constant regeneration, regulated by stem cells at the crypt base and supported by signalling pathways, including Wnt (Wingless and Int-1), Neurogenic locus notch homologue (Notch), epidermal growth factor (EGF) and bone morphogenetic protein (BMP) (24, 29, 43, 44). These pathways coordinate proliferation, differentiation, and lineage allocation of epithelial cells, maintaining homeostasis and barrier function (33, 34, 43, 44).

Weaning stress leads to a reduction in quiescent homeobox-only protein+ (HOPX^+^) and active sex-determining region Y-box 9 + (SOX9^+^) stem cells in piglets, particularly in the small intestine (34). This alteration in stem cell dynamics may influence susceptibility to *L. intracellularis*. During peak infection, Notch-1 is upregulated whilst WNT/β-catenin signalling is suppressed, promoting immature crypt cell hyperplasia and goblet cell depletion (18, 29, 35). Apoptosis and autophagy dysfunction also impair mucin production, contributing to mucosal thickening (45, 46).

**Figure 3:**
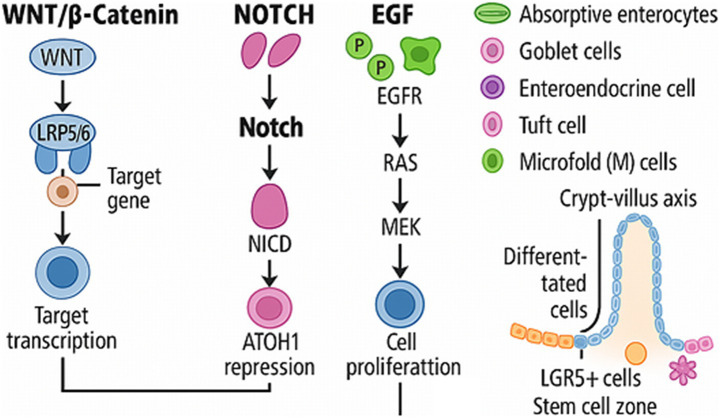
Overview of key signalling pathways regulating intestinal epithelial homeostasis. WNT, Wingless-related integration site family; LRP5/6, Low-Density Lipoprotein Receptor-Related Protein 5/6; NOTCH, Neurogenic locus notch homologue; NICD, Notch Intracellular Domain; ATOH, Atonal Homologue 1; EGF, Epidermal Growth Factor; EGFR, Epidermal Growth Factor Receptor; RAS, Rat Sarcoma; MEK, Mitogen-Activated Protein Kinase Kinase; LGR5^+^, Leucine-rich repeat-containing G-protein coupled receptor 5.

Wnt signalling sustains the stem cell pool and promotes proliferation (38, 39), Notch regulates absorptive versus secretory cell fate (18), EGF supports proliferation and migration, and BMP acts as a brake by promoting differentiation along the crypt–villus axis (40, 43, 44, 47). Disruption of this balance facilitates *L. intracellularis* colonisation and pathogenesis. Targeting these signalling networks may offer novel alternatives to antibiotics in managing PE by restoring intestinal epithelial homeostasis (31, 42). These changes in cell function and signalling are believed to hinder goblet cell development and promote the proliferation of crypt-immature cells. This process potentially leads to a decrease in mucin production and a thickening of the intestinal mucosa in cases of *L. intracellularis* infection (48). A visual overview of these pathways is provided in Figure 3. For a detailed understanding of the molecular pathways implicated in the host cell-pathogen interactions and the related immune response, we strongly recommend that the reader consult the detailed works published by Vannucci and Gebhart (29) and Obradovic and Wilson (49).

**Figure 4:**
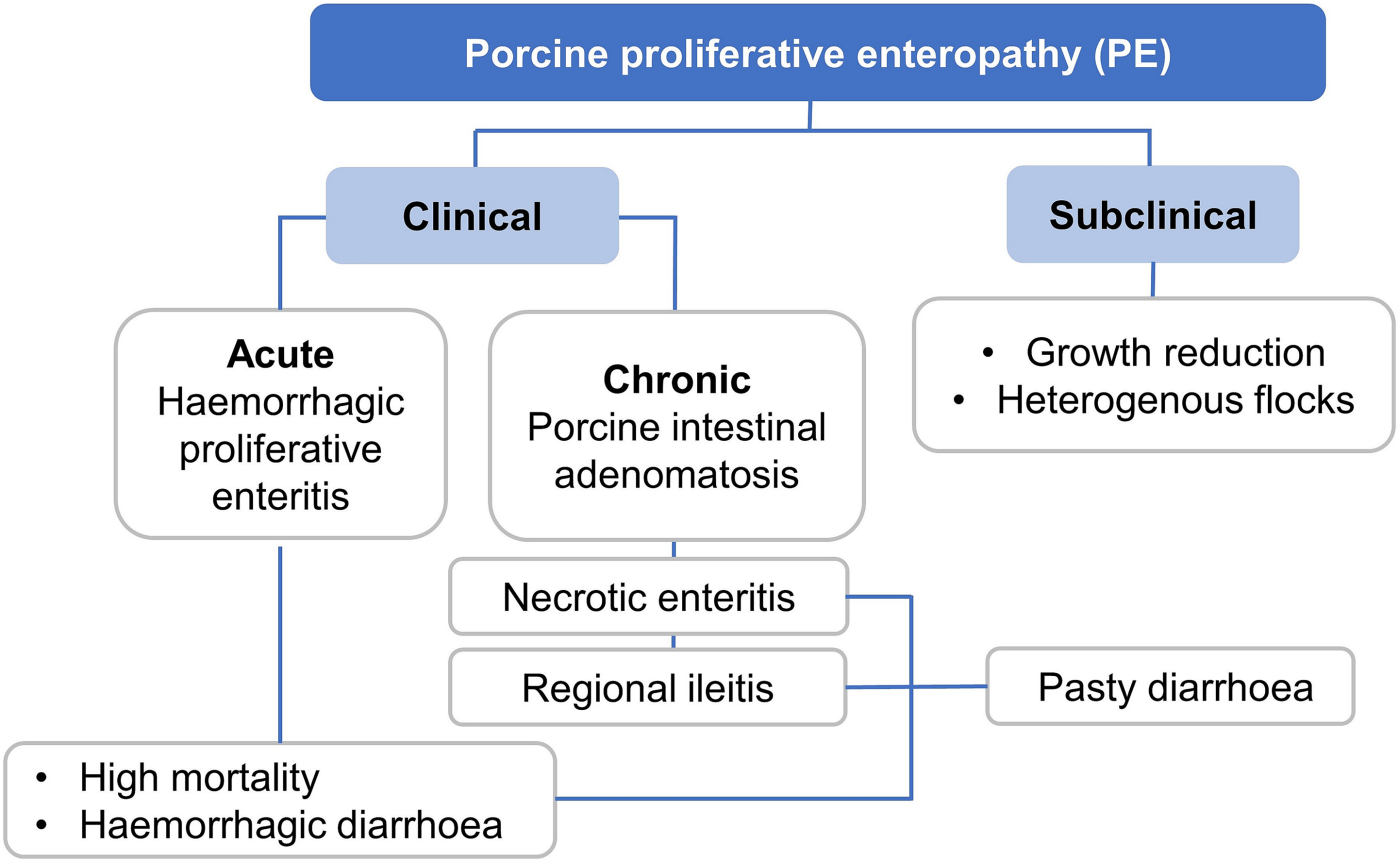
Classification of porcine proliferative enteropathy (PE) stages and main signs caused by *Lawsonia intracellularis*.

Key features of PE may vary and include an acute haemorrhagic form of diarrhoea, with the severity and colour of faeces varying, with occasional black tarry faeces to frank blood (50). The affected pig may demonstrate weakness and pallor, along with rapid death. In subclinical cases, a substantial variation in pig weights and sizes has been noticed with few sporadic diarrhoeas, decreased body weight gain, anorexia, and apathy (48). Emerging evidence suggests that chronic or subclinical *L. intracellularis* infections (whilst often clinically silent) can nonetheless impair growth performance, damage intestinal mucosa, and predispose animals to secondary infections, all contributing to a higher risk of carcass downgrading or condemnation. For example, it has been found that vaccinating subclinically infected pigs improved carcass quality, and vaccine-based reduction in intestinal lesions was also associated with better systemic health and slaughter outcomes (4). PE manifests in a variety of clinical forms, ranging from subclinical infections to acute and chronic disease presentations. The clinical form depends on factors such as pig age, immune status, environmental conditions, and bacterial load. Acute PE, also known as proliferative haemorrhagic enteropathy (PHE), typically affects older growers or finisher pigs and is characterised by sudden death and haemorrhagic diarrhoea with high mortality. Chronic forms include porcine intestinal adenomatosis (PIA), necrotic enteritis, and regional ileitis, leading to thickened intestinal mucosa and persistent diarrhoea, which may reduce performance over time. Subclinical infections, which are most common in grow-finish pigs, may go unnoticed but can significantly impact growth rates and flock uniformity. A schematic summary of the clinical spectrum of PE is shown in Figure 4.

## 5 Host-bacteria interaction: understanding the immune response against *Lawsonia intracellularis* in pigs

The actual cell receptor(s) involved and the actual bacterial ligand(s) present on *L. intracellularis* that interact with these cell receptors remain unclear, despite previous cell culture studies. The flagellar filament of bacteria, composed of numerous flagellin proteins, is detected by Toll-like receptors (TLR5) on epithelial cells, which trigger the Nuclear Factor kappa-light-chain-enhancer of activated B cells (NF-κB) and mitogen-activated protein kinase (MAPK) signalling pathways, thereby activating pro-inflammatory genes. Two flagellin proteins present on *L. intracellularis*, Lawsonia flagellin C (LfliC and LFliC), serve as pathogen-associated molecular patterns (PAMPs), have been identified in the interaction between the bacterium and its host, influencing the immune response, wherein the expression of flagellin protein specifically activated the NF-κB and MAPK pathways in human embryonic kidney epithelial cells. Thus, exploring the binding partners of cytoplasmic flagellin and uncovering the underlying mechanisms could provide valuable insights and is a promising area for future research (29, 51–54). Following invasion and intracellular replication within crypt epithelial cells, *L. intracellularis* antigens are processed by dendritic cells and macrophages, which then present them to T cells via MHC molecules in local lymphoid tissues. This activates a Type 1 T helper cell (Th1)-mediated immune response, characterised by Interferon-gamma (IFN-γ) production from CD4 + T cells and cytotoxic activity by CD8 + T cells (both critical in controlling intracellular infections) (51, 55, 56). IFN-γ further activates macrophages, enhancing their ability to clear infected cells.

Humoral immunity also plays a role. Although antibody responses lag behind cell-mediated responses, both systemic IgG and mucosal IgA have been observed following natural infection and vaccination. Secretory IgA contributes to mucosal protection by neutralising extracellular bacteria and inhibiting reinfection (52, 57, 58). Moreover, vaccination with live attenuated strains has been shown to induce both cellular and humoral immunity, protecting against reinfection (29, 51–54). In chronic or subclinical infections, the immune response may be insufficient to prevent bacterial persistence, leading to prolonged epithelial hyperplasia and disruption of intestinal barrier function. *L. intracellularis* infection has been associated with altered WNT/β-catenin and Notch signalling pathways in the intestinal crypts, affecting stem cell turnover, inhibiting goblet cell differentiation, and reducing mucin production This impairs mucosal defence and contributes to pathogenesis (29, 45, 55).

Recent transmission studies have shown that *L. intracellularis* can spread efficiently within pig populations, with a basic reproduction number (R₀) of 3.35 and a transmission rate of 0.096 per day, even when introduced by a single infected pig (59). These findings underscore the pathogen’s ability to persist and propagate under natural conditions, reinforcing the importance of early immune control and biosecurity. These mechanisms illustrate that the host immune response to *L. intracellularis* involves a tightly regulated interplay between innate sensors, pro-inflammatory signalling, T-cell-mediated immunity, and mucosal antibody responses (56). Disruption of these responses may allow chronic colonisation and disease progression, underlining the importance of integrated immune control and highlighting targets for vaccine and non-antibiotic-based interventions.

The immune response against *L. intracellularis* includes both humoral and cell-mediated immunity. Natural infection confers robust immunity, and specific cell-mediated immunity and IgA have been detected in the intestinal lumen of infected pigs (49, 51). *L. intracellularis* shows a unique challenge to the swine immune system. Like many other intracellular bacteria (such as *Mycobacterium* spp. and *Brucella* spp.), *L. intracellularis* has mechanisms to modulate the host’s immune system as it enters the cells via a delayed-type hypersensitivity (DTH) reaction. Studies have indicated a significant DTH response in infected intestines at 24 h post-cutaneous antigen sensitisation, with alterations in apoptosis reactions (31, 51). This DTH reaction was observed at a high concentration of 10^9^ *L. intracellularis* organisms. As an intracellular bacterium, it resides within the cells of the intestine, specifically targeting enterocytes (Figure 2). This localisation allows it to evade some aspects of the host’s immune surveillance. Understanding the immune response to this pathogen is critical for developing effective vaccines and therapeutic strategies. The immune response to *L. intracellularis* is complex, involving both the innate and adaptive arms of the immune system, each playing a crucial role in combating this infection (49).

The innate immune response is the first line of defence against *L. intracellularis*. Upon infection, innate immune cells such as macrophages and dendritic cells in the intestinal mucosa recognise the pathogen through pattern recognition receptors. This recognition leads to the secretion of various cytokines and chemokines, initiating an inflammatory response to contain the bacterium’s spread. The innate response also plays a pivotal role in shaping and informing the subsequent adaptive immune response. However, the effectiveness of the innate response is often challenged by the bacterium’s ability to survive and replicate within the host cells. It has been reported that *L. intracellularis* can survive and replicate within porcine macrophages (Figure 2C). This was observed through transmission electron microscopy, which revealed the bacteria within phagolysosomes and free in the macrophage cytoplasm, sometimes in binary fission. The study also employed qPCR to track bacterial numbers, demonstrating that *L. intracellularis* can proliferate at low levels within macrophages over time, suggesting a potential role for macrophages in the persistence and pathogenesis of the disease (60). Following the innate response, the adaptive immune system is activated. The involvement of both B cells and T cells characterises this response. T cells, particularly CD4 + T helper cells, play a crucial role in orchestrating a targeted immune response. They aid in activating macrophages and other immune cells to destroy the infected cells. Developing a robust adaptive immune response is critical to achieving long-term immunity against the bacterium (49). Future research on these aspects is key to developing more effective vaccines and therapeutic strategies. A deeper understanding of the immune response will also aid in managing PE, improving the health and productivity of swine herds worldwide (49, 61).

## 6 Diagnostic methods

The accurate diagnosis of proliferative enteropathy (PE) in swine remains a formidable challenge in veterinary medicine, primarily due to the multifactorial presentation of the disease and the complex biology of its etiological agent, *Lawsonia intracellularis*. The clinical spectrum of PE spans from subclinical manifestations to severe pathological states, encompassing chronic forms characterised by impaired growth performance and persistent diarrhoea, as well as acute presentations potentially culminating in mortality. The diagnostic process is further confounded by the presence of asymptomatic carriers and animals harbouring subclinical infections, which serve as silent reservoirs and contribute to the insidious propagation of the pathogen within herds (9).

Given these diagnostic intricacies, the early and precise identification of PE is paramount for the successful implementation of disease control strategies and the optimisation of herd health outcomes. A multifaceted diagnostic approach is therefore imperative, integrating detailed clinical assessments with targeted laboratory analyses, alongside a thorough appraisal of herd epidemiological data and husbandry practises. Amongst these, laboratory diagnostics (particularly molecular and serological assays) constitute the cornerstone for definitive confirmation of PE, enabling the differentiation between active infection and subclinical carriage. As such, comprehensive diagnostic protocols are essential for mitigating the impact of this economically significant enteric disease in commercial swine operations. Diagnostic methods for ileitis caused by *L. intracellularis* in pigs include both direct and indirect approaches. Indirect methods measure antibodies against the pathogen using tests such as indirect immunofluorescence (IFA), immunoperoxidase monolayer assay (IPMA) or enzyme-linked immunosorbent assay (ELISA) (9). Direct detection methods include immunohistochemistry and fluorescent *in situ* hybridisation (FISH), as well as polymerase chain reaction (PCR), on tissue and faecal samples, respectively. The reliability of PCR applied to faecal samples in detecting *L. intracellularis* may be compromised by the presence of inhibitory substances, including competing non-target DNA, bile salts, and bilirubin, which can interfere with the DNA amplification (polymerase activity/efficiency) (7, 62, 63). Additionally, in animals recently vaccinated with live attenuated *L. intracellularis* strains, transient faecal shedding of the vaccine organism can lead to false-positive PCR results. Therefore, laboratory findings (particularly PCR results) should be interpreted with caution in vaccinated animals, as current tests do not differentiate between vaccine and wild-type strains. Several methods can be employed: Conventional PCR, typically followed by agarose gel electrophoresis, remains a sensitive and specific method for detecting *Lawsonia intracellularis* DNA in faecal samples or intestinal tissue (64). This method can identify, in one step, the pathogen even in subclinical cases, making it invaluable for early detection (64). Nested PCR is more sensitive than conventional PCR (65). However, quantitative PCR (qPCR) (especially real-time TaqMan assays) has now become the gold standard, offering enhanced sensitivity, faster results, and the ability to quantify bacterial load (66).

Histopathological analysis allows the confirmation of lesions in intestinal tissue samples under a microscope and is considered a traditional and reliable method, and the primary way to reach a conclusive diagnosis (9). Characteristic histological lesions are the proliferation of enterocytes and the reduction of the number of goblet cells, which are indicative of PE. Immunohistochemistry (IHC) enhances histopathological diagnosis by using antibodies to detect the presence of *L. intracellularis* antigens in tissue samples (67). Serological tests, including enzyme-linked immunosorbent assay (ELISA) and immunoperoxidase monolayer assay (IPMA), measure the antibody response to the infection. Whilst helpful in assessing herd exposure, serology has limitations in acute diagnosis due to the time taken for antibody development (68).

Diagnosis of porcine PE represents a significant challenge due to the variability in its clinical expression and the biology of the causative agent. The broad range of manifestations of the infection (from acute haemorrhagic forms to chronic or subclinical presentations) makes clinical diagnosis unreliable in many cases. Additionally, *L. intracellularis* is shed intermittently, which limits the sensitivity of diagnostic methods such as PCR when samples are not collected at optimal times. Sample quality and handling further influence diagnostic outcomes, as degradation during transport or delayed processing can impair both molecular and histopathological analyses (69). The presence of live-attenuated vaccine strains in recently vaccinated animals may also complicate the interpretation of PCR and ELISA results (69).

Furthermore, co-infections with pathogens such as *Salmonella* spp. or PCV2 can obscure the clinical signs of PE or exacerbate them, requiring differential diagnosis. Importantly, subclinical infections are common and may significantly impact growth performance without obvious clinical signs, necessitating routine surveillance through serology or quantitative PCR (qPCR) (9). Despite these constraints, qPCR remains the gold standard for diagnosing *L. intracellularis* due to its high sensitivity, specificity, and ability to quantify bacterial load in faecal and tissue samples (69). It is currently impossible to differentiate between infected and vaccinated animals (DIVA). There are no markers in commercial *L. intracellularis* vaccines. Still, the development of an amplification method based on the genomic differences between *L. intracellularis* vaccine and wild-type strains would be an interesting approach for the development of a DIVA method (9).

Recent advancements in diagnostic technology are providing new avenues for detecting PE. Molecular techniques, such as quantitative PCR, offer more precise quantification of bacterial load, aiding in the understanding of the severity of infection (11). The most frequently utilised gene for targeting in diagnostics is the 16S ribosomal DNA (16S rDNA) gene (9). However, other markers, such as aspA99 and ubiE, have also been employed to identify *L. intracellularis* (64, 69). Several different primer pairs have been applied to the detection process of this bacterium. Additionally, metagenomic sequencing can support the usefulness of functional nutraceuticals and/or immunomodulator supplementation in reducing the impact of enteric pathogens and pathogen shedding rates in food animals without the use of antimicrobials, as well as identify co-infections, which are common in cases of ileitis and can impact treatment decisions (11). On-farm rapid tests are also being developed, offering quicker and more accessible diagnostics (9). However, accurate diagnosis still relies on integrating laboratory results with clinical signs and herd management history. Several diagnostic techniques are available for detecting *L. intracellularis* in pigs, each with distinct advantages and limitations that depend on the clinical context, tissue availability, and herd-level surveillance goals. Table 2 provides a comparative overview of commonly used diagnostic methods, summarising their sensitivity, specificity, turnaround times, and key practical considerations. Veterinarians play a crucial role in interpreting these findings to inform decisions about disease management and control strategies. It is also essential to consider the biosecurity measures and vaccination status of the herd, as these factors significantly influence the prevalence and manifestation of PE.

**Table 2:** Comparison of main diagnostic methods used for the detection of *Lawsonia intracellularis* in pigs.

Diagnostic method	Sensitivity	Specificity	Turnaround time	Sample type	Comments	References
PCR	Moderate–High	High	1–2 days	Faeces, intestinal tissue	Requires DNA extraction and gel electrophoresis	(8, 188)
qPCR	High	High	<24 h	Faeces, mucosal scrapings	Quantitative, rapid, and currently considered the gold standard	(66, 188)
IHC	High	High	2–3 days	Intestinal tissue	Useful post-mortem, confirms presence in enterocytes	(8, 76, 188)
ELISA	Moderate	Moderate	2–5 days	Serum	Indicates exposure, not active infection; useful in herd monitoring	(76, 188)
FISH	High	High	2–3 days	Intestinal tissue	Visual confirmation in tissue requires specialised equipment	(76, 188)
H&E	Variable	Low–moderate	2–3 days	Intestinal tissue	Supportive; may miss mild or chronic infections	(188)

PCR, Polymerase Chain Reaction; qPCR (Real-Time PCR), Quantitative Polymerase Chain Reaction; IHC, Immunohistochemistry; ELISA, Enzyme-Linked Immunosorbent Assay; FISH, Fluorescence In Situ Hybridisation; H&E, Haematoxylin and Eosin Staining.

## 7 Advantages and limitations of the use of antibiotics

The use of antibiotics in animal production has three primary purposes: therapeutic, preventive, and growth promotion. Therapeutic and preventive uses involve relatively high dosages for short periods of treatment and reduce the disease’s occurrence. Antibiotics used as growth promoters are administered at low (subtherapeutic) dosages as feed additives, decreasing morbidity and mortality and improving growth and the feed conversion rate (70–72). Thus, production costs are reduced, with 10–15% less feed required to achieve expected performance levels (73). One report on global trends in antimicrobial use in food animals projected that pigs will present the most considerable growth in antimicrobial consumption, contributing 45% to the total increase between 2017 and 2030 (74). Medication of older pigs (such as breeding stock) has not shown the potential to eliminate *L. intracellularis* infections. Partial depopulation and medication-based eradication attempts have been largely unsuccessful (48). Since PE can vary in onset time across different farms and between batches of pigs within the same farm, in-feed antimicrobials added too late may not adequately reduce clinical signs or improve performance (48, 75). Conversely, administering antimicrobials too early can hinder pigs’ exposure to the pathogen, preventing the development of natural immunity. As a result, these animals may remain immunologically naïve and be at greater risk of developing the acute haemorrhagic form (PHE) upon later exposure (76).

Misuse and extensive use of antibiotics can result in the development of resistance. Resistance can be acquired and transferred by commensal and pathogenic bacteria (77, 78). Resistance genes may also be transferred to retail meat products and the environment, thus spreading to animal and human populations. The extent to which this scenario contributes to an increased risk of therapeutic failure in humans is controversial (79), with most human-based resistance issues being ascribed to overuse amongst the human population rather than livestock. Several countries have developed policies to reduce or ban antibiotic use of antibiotics in animal production. For instance, the European Union (Reg. No. 1831/2003/EC) has prohibited the use of antibiotics as growth promoters in animals since 2006 (80). The U. S. Food and Drug Administration (FDA) began restricting medically necessary antimicrobials in 2017. Allowing only therapeutic purposes under veterinarian prescribing (81). Since 2020, China has banned all growth-promoting pharmaceutical feed additives, except traditional Chinese medicines, which will be withdrawn from animal production (82). Antibiotic-free animal products have experienced significant market growth and increased opportunities in recent years. Antibiotic resistance poses a worldwide threat to public health. The One Health approach is crucial for understanding and preventing the spread of antibiotic resistance from farm to table (83).

## 8 Non-antibiotic alternatives to control PE

### 8.1 Vaccines

Commercial vaccines against *L. intracellularis*, both live attenuated and inactivated, are available for prophylactic use, each with advantages and disadvantages. The development of such vaccines represents a significant advancement in swine health management. Vaccination against this pathogen has become a key strategy in preventing the economic losses associated with the disease, characterised by poor weight gain and diarrhoea. Two main types of vaccines are available: inactivated (killed) and live attenuated. Live attenuated vaccines have been particularly effective in inducing immunity in pigs, offering both ease of administration and long-lasting protection. The live attenuated vaccines for *L. intracellularis* were designed to provoke an immune response without causing the disease. This vaccine closely mimics a natural infection, thus providing robust and comprehensive immunity. It is typically administered orally, which is stress-free for the animals and mimics the natural route of infection, leading to greater localised immunity in the gut where *L. intracellularis* colonises. The immune response triggered by this vaccine involves both humoral and cellular immunity, providing a broad defence mechanism against the pathogen (51). Live bacterial vaccines may be antagonised by the simultaneous administration of certain antimicrobials; therefore, the live attenuated vaccine for *L. intracellularis* is often administered to suckling piglets at 2–3 weeks old via an oral drench or oral administration by gel onto the farrowing area (84, 85).

In contrast, inactivated vaccines contain killed bacteria and are usually administered via intramuscular injection. Inactivated vaccines can be administered using two distinct delivery systems: conventional intramuscular injection and intradermal application via needle-free devices. Whilst both routes aim to elicit protective immunity, intradermal vaccination tends to promote stronger local antigen presentation and has shown promising efficacy in reducing lesion severity and improving immune stimulation, particularly in inducing mucosal and cell-mediated responses (86, 87). Their effectiveness is considered more limited in terms of immunity range and duration than live attenuated vaccines (88, 89). Maternal antibodies might antagonise the effectiveness of injectable killed vaccines in piglets (52). The immune response induced by inactivated vaccines is predominantly humoral. Killed vaccines are usually formulated with various adjuvants to stimulate a more robust immune response (52, 88, 89). Combination vaccines for pigs, including *L. intracellularis* amongst other injectable antigens (such as porcine circovirus 2 and *Mycoplasma hyopneumoniae*), can help reduce the number of vaccine-related injections and manipulations (90). The piglet’s immune system is not considered sufficiently developed to receive vaccine administration before 2 weeks old (91).

Compared with vaccination, natural *L. intracellularis* infection induces a strong immune response involving both humoral and cell-mediated mechanisms. Mucosal immunity is characterised by the production of secretory IgA in the intestinal lumen, whilst systemic responses include DTH and elevated IFN-γ levels, which contribute to long-term immunity and control of re-infection (51, 92). Guedes et al. demonstrated a robust DTH response in pigs 24 h after cutaneous antigen sensitisation with *L. intracellularis*, suggesting a strong T-cell-mediated component in natural infection (92). Importantly, these DTH responses are considered protective rather than pathologic, as they reflect memory T-cell activation and effective host control of intracellular pathogens. This aligns with the broader understanding of DTH as a Th1-type response involving IFN-γ production, critical for bacterial clearance without inducing immunopathology (51).

In contrast, vaccination (whether live attenuated or inactivated) elicits a protective immune response that is often more targeted. Live oral vaccines reduce clinical signs, faecal shedding, and mortality, but may not always prevent colonisation or transmission (93, 94). Some studies have shown these vaccines elicit a measurable mucosal IgA and systemic immune response (52), although DTH reactions appear less pronounced compared to natural infection (93, 94). Moreover, intradermal inactivated vaccines have recently shown higher efficacy in reducing lesions and mortality but require further investigation to clarify their immunological mechanisms (86). Musse et al. further showed that intramuscular vaccination significantly reduced diarrhoea, antimicrobial use, and *L. intracellularis* shedding, whilst improving lean meat percentage in naturally infected Danish herds (86, 87). Vaccination may also influence gut microbiota composition, potentially contributing to reduced pathogen colonisation. It has been demonstrated that oral vaccination altered microbial communities in the gut, favouring beneficial species and decreasing *L. intracellularis* abundance (95). To better illustrate the differences in immune responses elicited by natural *L. intracellularis* infection compared to various vaccination strategies, Table 3 summarises key immunological parameters and associated outcomes for each approach.

**Table 3:** Comparative immunological responses and effects of natural *Lawsonia intracellularis* infection and vaccination strategies in pigs.

Parameter	Natural infection	Live oral vaccine	Inactivated intradermal vaccine	Intramuscular vaccine
DTH/IFN-γ response	Strong DTH and high IFN-γ response (51, 56)	↓ DTH response observed (56, 93)	Limited data; systemic responses not fully characterised (86)	Moderate systemic response; humoral immunity confirmed (87)
Mucosal IgA	Robust mucosal IgA production (51, 56)	↓ than natural infection (56, 93)	Induced mucosal immunity; mechanisms under study (86)	Moderate mucosal response observed (87)
Reinfection risk	Often sterile immunity post-infection (51, 56, 135)	↓ clinical signs; reinfection possible (93)	Superior lesion control; reinfection risk not fully eliminated (86)	↓ transmission and signs (87)
Faecal shedding	↓ naturally post-recovery (51)	↓ shedding ↓ shedding duration (93, 95)	↓ shedding vs. unvaccinated pigs (86)	↓ shedding duration (87)
Clinical signs and mortality	↑ mortality (if untreated) severe enteropathy (51)	↓ signs and mortality (93–95)	↓ in lesion scores and mortality (86)	↓ diarrhoea and antimicrobial use (87)
Growth and productivity	↓ ADG and FCE (51)	↑ ADG and ROI in field trials (93, 94)	↑ weight gain and lean meat yield (86)	↑ ADG vs. unvaccinated; FCE comparable (87)
Microbiota impact	Potential dysbiosis; altered mucosal environment (51)	Modulates microbiota towards beneficial taxa (↑ *Lactobacillus* and ↓ *L. intracellularis* abundance) (95)	No information	No information

DTH, Delayed-Type Hypersensitivity; IFN-γ, Interferon-gamma; ADG, Average Daily Gain; FCE, Feed Conversion Efficiency; ROI, Return on Investment.

### 8.2 Prebiotics and probiotics

The pig gut microbiota consists of a complex community of thousands of microbial species established soon after birth. Strategies that enhance beneficial bacterial populations are being increasingly explored as alternatives to antibiotics in managing enteric diseases, such as PE. Prebiotics are defined as non-digestible food ingredients that stimulate the growth of beneficial bacteria in the colon (96–98). Prebiotics are suggested to modulate the gut environment, making it less favourable for the proliferation of pathogens like *L. intracellularis* and more supportive of beneficial microbial populations such as *Lactobacillus*, *Bifidobacterium*, and *Faecalibacterium* spp. (99, 100). For instance, insoluble β-glucans from cereals such as barley and oats have been shown to increase *Lactobacillu*s and *Bifidobacterium* counts in the caecum and colon of pigs (100).

In a controlled feeding study, pigs fed oat-based diets exhibited significantly higher counts of these microbes in the caecum and colon compared to those on barley-based or enzyme-supplemented diets (101). Beneficial gut microbes may produce metabolites, such as short-chain fatty acids and bacteriocins, which influence the gut microbiota and immune responses (98, 102). In some cases, prebiotic non-digestible fibres may block pathogen adhesion to host cells, but this specific effect has not yet been demonstrated for *L. intracellularis* (69). Specific prebiotic feed additives include fructooligosaccharides (FOS), inulin, and mannooligosaccharides (MOS) (103), with other substances such as resistant starch and complex polysaccharides, including cellulose, hemicellulose, and pectin recognised for prebiotic activity (104). Trials adding distiller’s dried grains with solubles and soybean hulls to pig diets were associated with a slight reduction in *L. intracellularis* infection levels (73, 74). Supplementation of sow diets with short-chain fructooligosaccharides during late gestation and lactation improved measurable gut immune parameters and immune response against *L. intracellularis* in their offspring, with these piglets showing healthy gut morphology (96). The source of these benefits was not clear, but it may have involved an improved gut microbiota passed from sows to piglets, with more short-chain fatty acids present in the piglet gut (96). Although few studies directly investigate the control of *L. intracellularis* using prebiotics, the possible use of prebiotics in managing PE has been suggested (105, 106).

Probiotics consist of live microorganisms intended to be beneficial to gut health, such as isolates of *Lactobacillus*, *Bifidobacterium*, *Bacillus*, and *Enterococcus* spp., which are marketed for oral use in pig diets (107). These probiotics may work by competing with pathogenic bacteria for adhesion sites on the intestinal mucosa, producing antimicrobial substances, and enhancing the host immune response in the gut (108–110). Bacillus-based probiotics have gained considerable attention as viable alternatives to antibiotics in livestock, particularly due to their spore-forming ability, which ensures stability through feed processing and resilience in the gastrointestinal environment. Several strains such as *Bacillus subtilis*, *Bacillus licheniformis*, and *Bacillus pumilus* have been studied for their potential to improve gut health, enhance immune function, and reduce enteric pathogen loads in pigs and poultry (107, 108, 111, 112). One study suggested that administering *Bacillus pumilus* probiotics was associated with a reduced severity of clinical signs of PE in pigs and improved gut health (107). A major consideration of the usefulness of probiotics is gauging how many (if any) live bacteria administered into a pig’s diet actually make it through the farm feed preparation system and upper digestive tract to an intended site of colonisation in the lower bowel. The combined use of prebiotics and probiotics (synbiotics) has been suggested to function more effectively than either component alone (113). Current research is focused on identifying specific strains of probiotics and types of prebiotics that may be effective against enteropathogenic bacteria. Future directions also involve understanding the optimal dosages, timing, and administration methods for maximum efficacy (114).

Diet composition and physical form also influence microbial composition, subsequently the *L. intracellularis* infection in pigs (115, 116). Coarse, non-pelleted feed may reduce the prevalence of pathogens such as *L. intracellularis* and promote beneficial microbes including *Prevotella* and *Lactobacillus* spp. (115). Diet composition in terms of feed form may also influence infection dynamics. Pelleted diets have been linked to increased *L. intracellularis* colonisation and faecal shedding in pigs due to shifts in gut microbiota and reduced fermentation by-products like butyrate and acetate. Pelleted feed exhibited a higher pathogen load compared to meal-form diets (116). A study demonstrated that pigs fed coarse, non-pelleted diets exhibited a significantly lower burden of *L. intracellularis* in the ileal microbiota, suggesting that feed texture can modulate pathogen colonisation through its impact on microbial community structure (115). Additionally, it has been reported that fermented liquid diets delayed shedding and reduced intestinal lesions, highlighting feed form as a significant factor in subclinical ileitis management (117). However, research on these aspects remains scarce, and more research in this field is still necessary.

### 8.3 Phytogenics

Feed additives based on mixtures of phytomolecules, known as phytogenic (also referred to as phytobiotics or botanicals), have garnered significant attention in mainstream livestock health and nutrition trends (118, 119). These plant-based products, including essential oils and other botanical extracts, have been recognised for their beneficial effects on pigs’ growth performance, nutrient digestibility, biochemical profile, gene expression, hypocholesterolaemia, immunity, meat quality, fatty acid composition, amino acid content, and especially in mitigating the impact of disease and environmental stressors on pig gut health, mainly due to antimicrobial, anti-inflammatory, and immunomodulatory properties (110, 120, 121). The metabolism of essential oils and other plant extracts follows different enzymatic degradation pathways *in vivo*, making it essential to study both their chemical profiles and metabolites. Biotransformation of phytomolecules through phase 1 (oxidation, reduction, hydrolysis) and phase 2 metabolism is crucial for assessing their safety profiles (122). Although various pharmacodynamic properties of different secondary plant metabolites have been reported *in vitro*, their actual availability in target organs remains unverified. Hence, research on absorption, distribution, metabolism, and excretion is essential to bridge the gap between *in vitro* and *in vivo* findings (82, 83). Due to the complexity of their chemical composition, volatility, and susceptibility to metabolic degradation, understanding their bioavailability, pharmacokinetic, and pharmacodynamic parameters is critical for their effective and practical application in livestock health (123). Limitations on the use of antibiotics in livestock farming have led to a search for potentially valuable phytomolecules as replacements (124), including those intended for use in PE (125–127). Several plant secondary metabolites have demonstrated antibacterial activity, which can disrupt bacterial cell walls and interfere with their metabolic processes, reducing the pathogen load in the intestines of pigs (119). Incorporating some phytogenic feed additives into feed was observed to reduce the incidence and severity of PE, offering a non-antibiotic alternative for therapy (125–127). Beyond their antimicrobial action, phytogenics are also known for their antioxidant and anti-inflammatory properties (126, 128–130). Their use in gastrointestinal diseases, such as PE, may lead to situations where the product enhances the immune response by facilitating efficient pathogen clearance and reducing the likelihood of severe infection (131).

A broad spectrum of phytomolecules has been demonstrated to have a range of properties relevant to animal health, including antimicrobial, antioxidant, anti-inflammatory, and immunomodulatory properties (132–134). The possible efficacy of orally administered phytogenics may therefore be greater in gastrointestinal tract conditions. Phytogenic preparations containing extracts of plants such as chestnut (*Castanea sativa*), oregano (*Origanum vulgare*), thyme (*Thymus vulgaris*), coriander (*Coriandrum* sp.), garlic extracts (*Allium sativum*) and plume poppy (*Macleaya cordata*) have shown initial promising results as antibiotic alternatives for the control of PE (86, 95, 96, 135). Feeds supplemented with isoquinoline alkaloids derived from *Macleaya cordata* extract have been shown to mitigate intestinal lesions caused by *L. intracellularis* and to reduce the incidence of carcass condemnation at slaughter, suggesting an improvement in systemic health (136). For instance, it was evidenced that weaned piglets fed a diet supplemented with *Macleaya cordata* extract and benzoic acid exhibited improved growth performance, enhanced villus height and villus-to-crypt ratios, elevated antioxidant enzyme activities, and beneficial shifts in gut microbiota, including increased *Lactobacillus* and reduced *Escherichia* and *Shigella* populations (137). Recent evidence supports the use of phytogenic-based feed additives in managing co-infections relevant to porcine enteric diseases. For example, a recent study demonstrated that a phytogenic blend administered through feed significantly reduced clinical signs, lesion severity, and pathogen load in pigs co-infected with *L. intracellularis* and *Brachyspira hyodysenteriae*. Their findings further indicated improved gut histomorphology and reduced inflammatory markers, underscoring the potential of phytogenics as an effective non-antibiotic strategy to control multiple enteropathogens in swine production systems (138). Therefore, improvements in intestinal and systemic health through phytogenic supplementation may reduce these indirect effects and support antibiotic-reduction strategies in swine production (139).

Challenges remain related to the significant variations and determination of the composition and concentration of active ingredients and secondary metabolites in source plant materials and any phytogenic products derived for use in animals. Batches of unpurified phytogenic products may suffer significant variations due to geo-climatic factors, physiological variations, soil quality, agricultural practises, extraction, fabrication, and storage processes (118). Identifying active compounds in phytogenic feed additives is essential for understanding their mode of action (129). Therefore, standardisations of plant-derived feed additives are necessary to guarantee the minimum or range of concentration of suggested active compounds in any commercial product that impacts animal health and productivity (118). Achieving consistent quality and quantity of natural phytogenic feed additives requires optimised growing conditions, appropriate harvest timing, genetic engineering (140, 141), as well as regular quality control of raw material and end products. The stability of phytogenic feed additive compositions and their biological activity, particularly essential oils, can also be hindered by heat, light, metals, the feed matrix, and the availability of water and oxygen in the production system (142).

Commercial plant-derived products should have available data on their plant chemotype, chemical composition and relevant field and challenge exposure studies, matching those for antibiotics or other pharmaceutical treatments (118, 124, 143). Whilst the use of plant-derived bioactive molecules in controlling PE and other intestinal diseases shows promise, further research is needed to fully understand their pharmacokinetics and mechanisms of action, as well as to optimise their application in swine production. Studies focusing on identifying the most effective phytotherapeutic preparation compounds, including phytogenic feed additives, determining optimal dosages, and understanding their interactions with other dietary components are essential for this future-oriented field of research for sustainable livestock production (118).

### 8.4 Other chemicals

Niacin (nicotinamide, vitamin B3) may influence the function of neutrophils and macrophages within the innate immune system (144), potentially aiding in the more effective clearance of pathogenic bacteria and viruses (145, 146). Nicotinamide has been reported to exert its anti-inflammatory action, in part, by suppressing neutrophil chemotaxis (147). The potential application of niacin as a control strategy for gut infections in pigs presents its own challenges. Determining the mechanism of action, optimal dosage, and delivery method for the overall health of swine is critical. Niacin remains a possible non-antibiotic candidate, warranting further research and consideration in disease management (148, 149).

Lysozyme is a muramidase enzyme, which is naturally present in tears, saliva and milk (150, 151). Lysozyme can disrupt the cell walls of bacteria, exerting a bacteriolytic effect (150). Lysozyme combats bacteria through multiple mechanisms: it disrupts the peptidoglycan layer of Gramme-positive bacteria, making some bacteria more susceptible to antimicrobials and osmotic stress, whilst Gramme-negative bacteria are more resistant due to their outer membranes (151). At high concentrations, lysozyme exhibits a non-enzymatic antimicrobial activity by disrupting bacterial membrane integrity or triggering the release of bacterial autolytic enzymes (152). Furthermore, lysozyme has immunostimulatory properties, enhancing antibody production, hypersensitivity responses, and disease resistance, with heat treatment potentially boosting these effects. These properties underscore its role in the innate immune system (153). Its application in swine health may utilise its ability to target and reduce the bacterial load in the intestines (151, 154). The optimal dosage and understanding of the long-term effects of dietary lysozyme supplementation in pigs are crucial areas of ongoing research. Additionally, the effectiveness of lysozyme against *L. intracellularis* specifically, and its interaction with other components of the pig’s diet and microbiota, requires further investigation. Future studies are also needed to explore the potential of lysozyme in combination with other therapeutic agents, such as probiotics or phytogenics, as part of an integrated approach to PE management.

Antimicrobial peptides (AMPs), or host defence peptides, are a critical immune mechanism and barrier against pathogenic bacterial invasion. Mature AMPs usually contain 12–100 amino acid residues, an amphiphilic molecular structure, and a positive charge, which optimises their interaction with cell membrane targets (155). AMPs can have a broad-spectrum antibacterial activity, representing potent effector molecules in the innate immune system. AMPs have antimicrobial, antiviral and antitumor effects and exhibit substantial *in vivo* effects, such as anti-inflammatory response, recruiting immune cells, promoting epithelial damage repair, and promoting phagocytosis of bacteria (155). However, few AMPs have entered the market to replace antibiotics. Limitations in their use include the complexity and high costs of their production as pharmaceutical agents, particularly for animals. The AMP molecules’ efficacy requires a complete 3-D structure, which is difficult and expensive to manufacture. AMPs also suffer from high metabolic instability, so dosage and delivery may be difficult to achieve. Even so, AMPs have been suggested as a potential strategy for controlling bacterial pathogens in the swine industry (156–159).

Another emerging field in animal and veterinary sciences is nanobiotechnology, offering various practical applications, including therapeutic, diagnostic, and nutritional uses. Nanoparticles (NPs), in general, are particles in size < 100 nm that can enter cells, tissues and organs and are recognised for their antibacterial, antifungal, antiviral, antiprotozoal, and antioxidative properties (160–163). NPs are employed to meet the animal’s requirements for elements, enhance their productivity, improve microbial profiles and immune status, as well as diminish the risk of diseases. For example, silver, copper, selenium, and zinc nanoparticles can serve as alternative health and growth-promoting additives to antibiotics (160, 163–165). It is essential to acknowledge that metal nanoparticles may enhance cellular uptake and distribution throughout an animal’s body, which could impact their toxicity (166, 167). Nanoparticles in an animal’s diet can trigger inflammation or even result in cell death, leading to pathological changes in various organs, including the liver, pancreas, kidneys, small intestine, adrenal glands, and brain. Therefore, additional research is crucial to confirm that the addition of metal-containing nanoparticles to animal nutrition is safe and does not have a negative impact on humans, animals, or the environment (164).

## 9 Biosecurity, hygiene and husbandry practises

Good hygiene and husbandry practises are essential in reducing the risk of infection and spreading PE and other enteric pathogens within a swine herd. This includes strict access control, with biosecurity protocols, regular cleaning and disinfection of pens, feeding areas, and equipment to minimise the presence of the pathogen in the environment (168). Proper waste management and control of rodent and insect populations appear also to be important, as they can be vectors for disease transmission (23, 28). Implementing strict external and internal biosecurity measures, such as controlling farm access and using area-specific protective clothing and boots, can further help in reducing the introduction and spread of the pathogen (48).

The type of production system—whether a closed-cycle (farrow-to-finish) or multi-site (three-site or two-site) production model—also plays a significant role in PE risk and management. In closed-cycle systems, where all stages of production occur on a single site, the potential for continuous exposure to *L. intracellularis* may be higher if strict internal biosecurity and thorough sanitation between age groups are not rigorously maintained. However, this system also facilitates tighter control over pig flow and staff management (10). In contrast, multi-site systems (e.g., separate nursery and grow-finish units) can reduce cross-contamination between age groups but may introduce additional risk through frequent animal transport and environmental transitions, which are known stressors that predispose pigs to PE. The choice of system influences batch management strategies, particularly all-in-all-out (AIAO) protocols (169). Proper batch separation and downtime between groups are easier to enforce in well-structured multi-site systems, thereby reducing persistence of infections. In both systems, the strict application of biosecurity measures, sanitation protocols, and animal flow control is critical to mitigate the risks associated with PE transmission and outbreaks (10).

Alongside hygiene, effective husbandry practises are crucial in managing PE. This includes management strategies such as all-in-all-out production systems, which involve housing pigs of the same age group together and thoroughly cleaning and disinfecting the facilities between groups. Such systems help break the cycle of infection and reduce the level of exposure of young, susceptible pigs to the pathogen. Nutritional management also plays a key role, with diets tailored to support gut health and immunity being particularly beneficial. Optimal housing and ventilation conditions, appropriate stocking densities, and minimising stress-inducing practises such as heat stress are essential components of effective PE control (76, 170). Improved farm hygiene measures will reliably reduce the prevalence and severity of PE (168). Quaternary ammonium-based compounds have effective anti-*L. intracellularis* disinfectant activities, but isolates appeared somewhat resistant to phenolic or iodine-based mixtures (21). The effectiveness of various commercial disinfectants against *L. intracellularis* was determined *in vitro*. Besides, certain disinfectants, including quaternary ammonium and their combinations with aldehydes, as well as oxidising agents, were highly effective at inactivating *L. intracellularis* under simulated conditions, including the presence of hard water and organic materials. The study suggests that these disinfectants could be reliable options for controlling the spread of *L. intracellularis* in swine farms, reducing the risk of PE (171).

Thoroughly washing and cleaning pig pens, facilities, boots, and equipment, along with effective rodent control on both single-site and multi-site farms, are likely to be more effective strategies for reducing PE (20, 27, 28, 172). These methods are generally more reliable than relying solely on slatted floors and sunken pits for faeces removal.

Regular monitoring and evaluation of health status, growth performance, and incidence of PE signs in the herd can provide valuable insights into the effectiveness of the implemented practises. Training farm staff in proper hygiene and animal handling techniques is also crucial for maintaining a consistently high standard of care. Additionally, collaboration with veterinarians for regular health check-ups and implementation of vaccination programmes can complement hygiene and husbandry practises in controlling PE. By adopting a comprehensive and proactive approach to hygiene and husbandry, swine producers can significantly reduce the prevalence and impact of PE, thereby enhancing their herds’ overall health and productivity.

**Figure 5:**
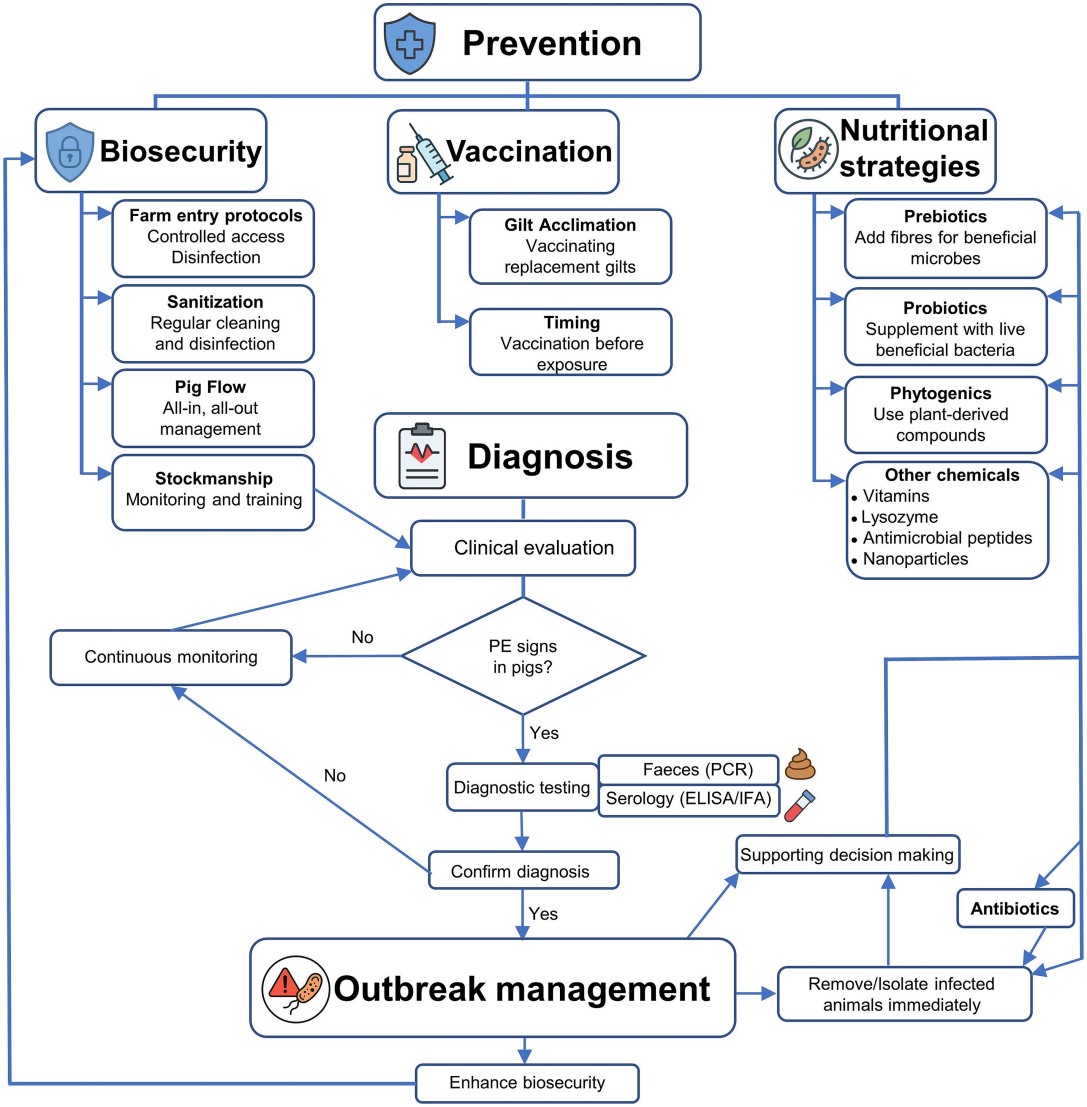
Decision-tree workflow for the prevention, diagnosis, and management of porcine proliferative enteropathy (PE).

To effectively address the complex aetiology and management of porcine PE caused by *L. intracellularis*, an integrated decision-tree model was developed to guide veterinarians, swine producers, and researchers through a structured approach to prevention, diagnosis, and outbreak management (Figure 5). The model is divided into three main stages: Prevention, Diagnosis, and Outbreak Management. In the Prevention stage, core strategies include biosecurity measures (e.g., sanitation, pig flow, staff training), vaccination protocols (including gilt acclimation and optimal timing), and nutritional interventions (such as the use of prebiotics, probiotics, phytogenics, and emerging technologies like nanoparticles). If clinical signs arise, the model progresses to the diagnosis phase, which integrates symptom recognition with laboratory-based diagnostics using faecal and blood samples. Upon confirmation of PE, the model advances to Outbreak Management, emphasising enhanced biosecurity, targeted treatment, and supportive decision-making to contain disease spread and reduce recurrence. This framework aims to guide veterinarians and farm managers in minimising PE impact through proactive and evidence-based actions.

## 10 Economic impacts

The dominance of the immature form of proliferating crypt epithelial cells in PE, with their characteristic morphology and staining, means that fewer membrane transporters related to digestion and nutrient acquisition are operative (carbohydrates, amino acids, lipids and Vitamin B12). Thus, reduced nutrient absorption by the immature intestinal mucosa is the primary and significant cause of the reduction in weight gain and feed conversion efficiency seen in PE-affected pigs (173).

PE, therefore, remains a significant economic concern in the swine industry due to its impact on animal health and farm productivity. The disease affects pigs by causing poor growth, increased feed requirements per kilogramme of gain, a higher percentage of lightweight pigs, and increased mortality rates (174). These issues create a bottleneck in swine production systems, affecting the overall economic efficiency and reducing the supply of breeding animals (174). Subclinical infections of PE are highly prevalent, leading to reduced production parameters, such as weight gain, feed conversion, and uniformity amongst pigs, but without apparent signs like diarrhoea or weight loss (175). This form of the disease can go undetected, still causing economic losses, but without overt signs of clinical illness.

The disease is responsible for poor feed conversion and a 6–20% reduction in average daily gain, resulting in increased days to market and greater variation in end weight. Pig farms affected by ileitis often experience performance setbacks such as poor feed conversion, increased days to market, and greater variability in end weights; elevated mortality is primarily limited to acute haemorrhagic cases (15). These statistics underline the critical importance of managing and preventing ileitis to minimise economic losses in the swine industry. In monetary terms, the financial losses due to ileitis are substantial. In 2005, economic losses due to PE in North American and European commercial production systems, particularly intensive grow-finish operations where *L. intracellularis* is endemic, were estimated from adverse impacts on slaughter weight, feed conversion efficiency, space utilisation, breeding problems and morbidity-mortality effects, totalling from USD 1 to USD 5 per affected growing pig (175). The impact is likely higher since those published estimates were based on clinical cases and did not include subclinical cases. There are also costs associated with diagnostics, hygiene, and medical interventions. Additionally, assuming trends of inflation and price increases, the mentioned costs are considerably higher today. More recently, in the United States, it was estimated that the disease causes a financial loss of around USD 4.65 per fattening pig, which amounts to an annual loss of USD 56.1 million for American pig farmers (176). Similarly, in Europe, the cost associated with PE can be up to 5 euros per pig, with the primary economic losses attributed to reduced average daily gain, poorer feed conversion efficiency, and increased mortality in growing and finishing pigs (177). Whilst PE primarily affects young pigs, subclinical infections may contribute to reduced performance consistency within herds. Recent study using modelling has highlighted the substantial economic burden posed by PE in commercial swine operations. The modelling report estimated that productivity losses in finishing pigs affected by *L. intracellularis* range from USD 5.98 to USD 17.34 per animal, depending on the clinical severity and herd management conditions (178). These losses stem primarily from reduced average daily gain, poorer feed conversion efficiency, increased morbidity, and carcass downgrading at slaughter. The analysis underscores the importance of early detection and strategic intervention (via vaccination, nutritional optimisation/supplementation, or antimicrobial protocols) to mitigate subclinical disease impact and preserve profitability across intensive production systems (178).

Live and inactivated vaccines are now widely implemented for the control of PE (29, 86). Their use is particularly critical in nucleus herds and during the introduction of replacement breeding animals into commercial operations (179). Historical reliance on acclimation protocols or timed medication regimens (without consistent vaccination) has proven insufficient. In such cases, naïve gilts, especially those transported to multiplier or satellite farms, have remained vulnerable and have been involved in significant PE outbreaks amongst breeding stock (179, 180).

## 11 Conclusion

In conclusion, PE poses a significant challenge in the swine industry due to its direct impact on animal health and productivity, as well as the emerging issue of antimicrobial resistance. The complexity of porcine PE, characterised by the diverse clinical signs caused by *L. intracellularis*, necessitates a multifaceted approach to management and control. With increasing concern over antimicrobial resistance, the traditional reliance on antibiotics is no longer a sustainable or effective long-term solution, highlighting the urgent need for integrated disease management strategies. Promising strategies include improved biosecurity, vaccination programmes, and the adoption of antibiotic alternatives such as prebiotics, probiotics, phytogenics, and immunomodulatory compounds. In particular, understanding the intestinal immune dynamics and crypt–villus interactions can inform targeted interventions against *L. intracellularis*. Future efforts should focus on standardising non-antibiotic interventions, validating strain-specific effectiveness, and improving diagnostic tools for field use. In light of the multifaceted nature of PE, an integrated, evidence-based management strategy that prioritises early diagnosis, tailored vaccination, and gut health support is the most promising route to sustainable control. Further research should prioritise the development of cross-protective vaccines, microbiota-focused interventions, and standardised phytogenic applications to reduce reliance on antibiotics.
